# Coordinate-based (ALE) meta-analysis of acupuncture for musculoskeletal pain

**DOI:** 10.3389/fnins.2022.906875

**Published:** 2022-07-22

**Authors:** Guodong Ha, Zilei Tian, Jiyao Chen, Shuo Wang, Aga Luo, Yunyu Liu, Juan Tang, Ningyuan Lai, Fang Zeng, Lei Lan

**Affiliations:** ^1^Acupuncture and Tuina School, The 3rd Teaching Hospital, Chengdu University of Traditional Chinese Medicine, Chengdu, China; ^2^Acupuncture and Brain Science Research Center, Chengdu University of Traditional Chinese Medicine, Chengdu, China; ^3^Key Laboratory of Sichuan Province for Acupuncture and Chronobiology, Chengdu, China

**Keywords:** musculoskeletal pain, acupuncture, functional magnetic resonance imaging, activation likelihood estimation, meta-analysis

## Abstract

**Background:**

Neuroimaging studies have been widely used to investigate brain regions' alterations in musculoskeletal pain patients. However, inconsistent results have hindered our understanding of the central modulatory effects of acupuncture for musculoskeletal pain. The main objective of our investigation has been to obtain comprehensive evidence of acupuncture for musculoskeletal pain diseases.

**Methods:**

The PubMed, Web of Science, Google Scholar, Embase, China National Knowledge Infrastructure (CNKI), VIP Database, China Biology Medicine disc Database, Clinical Trial Registration Platform, and Wanfang Database were searched for neuroimaging studies on musculoskeletal pain diseases published from inception up to November 2021. Then, the relevant literature was screened to extract the coordinates that meet the criteria. Finally, the coordinate-based meta-analysis was performed using the activation likelihood estimation algorithm.

**Results:**

A total of 15 neuroimaging studies with 183 foci of activation were included in this study. The ALE meta-analysis revealed activated clusters in multiple cortical and sub-cortical brain structures in response to acupuncture across studies, including the thalamus, insula, caudate, claustrum, and lentiform nucleus.

**Conclusions:**

The studies showed that acupuncture could modulate different brain regions, including the thalamus, insula, caudate, claustrum, and lentiform nucleus. The findings offer several insights into the potential mechanisms of acupuncture for musculoskeletal pain and provide a possible explanation for the observed clinical benefit of this therapy.

**Systematic review registration:**

https://www.crd.york.ac.uk/prospero/display_record.php?RecordID=227850, identifier: CRD42021227850.

## Introduction

Musculoskeletal pain disorders are a group of diseases characterized by nociception in the musculoskeletal system (muscles, ligaments, joints, and tendons), which include but are not limited to neck pain, low back pain, and fibromyalgia (Skootsky et al., 1989; Wolfe et al., 1995; Lawrence et al., 1998; Melhorn, 2014). Nowadays, musculoskeletal pain is a significant complaint. Adults with musculoskeletal pain account for 40.4–69.3% of the population (Abdulmonem et al., 2014). A potential peak of sufferers is anticipated as the population ages and young people adopt improper postures (Yuan et al., 2016). Musculoskeletal pain substantially impacts patients' quality of life and causes considerable societal and economic burdens since it limits daily activities and reduces productivity. On the other hand, the long-term usage of drugs is not indicated in clinical practice due to adverse effects (Ussai et al., 2015). As a result, alternative therapies should be given more consideration.

As an efficacious treatment for musculoskeletal pain, acupuncture has a long history of relieving the symptoms of such diseases in the East (Lenoir et al., 2020). It has been recommended as a positive therapy in a clinical practice guideline from the American College of Physicians (Qaseem et al., 2017). Acupuncture has been used to treat many types of musculoskeletal pain diseases (Mu et al., 2020; Zhang and Wang, 2020). However, we still have a limited understanding of how acupuncture works.

The current study showed that brain changes in musculoskeletal pain are widespread and involve the pain network as well as sensory, emotional, and cognitive control networks that process information (Mitsi and Zachariou, 2016). Various functional neuroimaging techniques have been used to ascertain which brain areas are metabolically activated or deactivated. That would help understand acupuncture analgesia from a central nervous system view. In previous studies, acupuncture has been found to regulate abnormal neural activities of the “pain matrix,” mainly the second-order (including posterior parietal, prefrontal and anterior insular areas) and third-order (including the orbitofrontal and perigenual/limbic networks) matrices responsible for pain memory in musculoskeletal pain sufferers (Apkarian et al., 2005; Borsook et al., 2010a; Garcia-Larrea and Peyron, 2013). After extensive literature research, the pain memory matrices, including the insula cortex (IC), inferior frontal cortex, thalamus, anterior cingulate cortex, medial prefrontal cortex (MPFC), and others, were mainly associated with acupuncture analgesia (Henry et al., 2011; Farmer et al., 2012; Chae et al., 2013; Villarreal Santiago et al., 2016). Although these neuroimaging studies have identified several brain regions activated or deactivated by acupuncture, the results still have to be appropriately interpreted. Simultaneously, we found that previous studies have failed to consider heterogeneity due to different imaging modalities (e.g., fMRI, PET, SPECT), study populations, experimental paradigms, and data analysis pipelines. Therefore, further studies are still necessary.

As mentioned above, activation likelihood estimation (ALE) is an excellent method for dealing with heterogeneity. It has been the primary method for integrating neuroimaging data in meta-analyses. Instead of calculating the presence or absence of brain activity in the region of interest, ALE analysis combines the study coordinates of all individuals to determine the statistical probability of brain regions being activated or deactivated. An ALE analysis is required due to the lack of consistent evidence in the current study. This analysis can help us better summarize acupuncture's significant modulatory effects on musculoskeletal pain.

This study aimed to investigate the brain activities of patients with musculoskeletal pain to obtain sufficient evidence of acupuncture's effectiveness in treating musculoskeletal pain diseases. To this end, this study first searched relevant databases, then evaluated the included literature. Finally, a comprehensive conclusion of how acupuncture treats musculoskeletal pain diseases was drawn by combining the ALE analysis results.

## Materials and methods

### Literature search and selection

Studies were obtained from the following databases: PubMed, Web of Science, Google Scholar, Embase, China National Knowledge Infrastructure (CNKI), VIP Database, China Biology Medicine disc Database, Clinical Trial Registration Platform, and Wanfang Database, searched from inception to November 2021. The relevant references from the retrieved papers have been added to the database for this study. Only whole-brain studies published in English were eligible for the review. Table 1 shows search strategies to replicate the other databases' selection processes. In addition, references to studies included in the review and clinical trial databases were manually screened to avoid omitted studies. The present meta-analysis was registered in PROSPERO (no. CRD42021227850).

**Table 1:** Searching strategy.

1.1 PubMed searching strategy	1.2 CNKI searching strategy
#1 musculoskeletal pain (MeSH Terms)	#1肌肉骨骼痛 （主题词）
#2 musculoskeletal pain (All Fields)	#2肌肉骨骼疾病 （主题词）
#3 musculoskeletal disease (All Fields)	#3肌肉痛 （主题词）
#4musculoskeletal disorders (All Fields)	#4肌肉疼痛 （主题词）
#5muscular diseases (All Fields)	#5骨骼痛 （主题词）
#6chronic musculoskeletal pain (All Fields)	#6关节炎 （主题词）
#7musculoskeletal Conditions (All Fields)	#7关节痛 （主题词）
#8muscle pain (All Fields)	#8关节疾病 （主题词）
#9Myalgia (All Fields)	#9纤维肌痛 （主题词）
#10myofascial Pain (All Fields)	#10肌筋膜疼痛 （主题词）
#11Fibromyalgia (MeSH Terms)	#11肩痛 （主题词）
#12neck pain (MeSH Terms)	#12腰痛 （主题词）
#13Osteoarthritis (MeSH Terms)	#13背痛 （主题词）
#14Arthritis (MeSH Terms)	#14颈痛 （主题词）
#15Arthrosis (MeSH Terms)	#15颈椎痛 （主题词）
#16Arthralgia (MeSH Terms)	#16#1OR#2 OR#3 OR#4 OR#5 OR#6 OR#7 OR#8 OR#9 OR#10 OR#11 OR#12 OR#13 OR#14OR#15
#17Joint Diseases (MeSH Terms)	#17功能性磁共振 （主题词）
#18Low Back Pain (MeSH Terms)	#18功能磁共振 （主题词）
#19Lumbago (MeSH Terms)	#19 fMRI主题词
#20Back Pain (MeSH Terms)	#20#17OR#18 OR#19
#21Backache (MeSH Terms)	#21针刺 （主题词
#22Shoulder Pain (MeSH Terms)	#22穴位 （主题词
#23Cervicalgia (MeSH Terms)	#23电针 （主题词）
#24#1OR#2 OR #3 OR #4 OR #5 OR #6OR#7OR #8 OR #9 OR #10 OR #11 OR#12 OR#13OR #14 OR#15 OR #16 OR #17 OR #18 OR #19 OR #20 OR #21 OR #22 OR #23	#23电针 （主题词）
#25 acupuncture (MeSH Terms)	#24#21OR#22 OR#23
#26 acupuncture Therapy (MeSH Terms)	#25 Final search terms: #16AND #20 AND #24
#27 acupoint (MeSH Terms)	
#28 acupuncture Point (MeSH Terms)	
#29 electroacupuncture (MeSH Terms)	
#30 electro-acupuncture (MeSH Terms)	
#31#25OR#26 OR#27 OR#28 OR#29 OR#30	
#32Functional magnetic resonance imaging (MeSH Terms)	
#33 Functional MRI (MeSH Terms)	
#34 fMRI (MeSH Terms)	
#35#32 OR#33 OR#34	
#36 Final search terms: #24AND #32 AND #35	

### Inclusion criteria

1. The study must be performed for whole-brain analysis by fMRI.
2. The research results had to be presented in Talairach or MNI coordinates.
3. Documents presented by the same research team must use different raw data.
4. Cohort studies and randomized controlled trials were included only if neuroimaging results were available.

### Exclusion criteria

1. Area-of-interest scans, hyper-scanning, and small-volume-correction studies were excluded.
2. Other secondary research such as conference articles, reviews, animal experiment articles, case reports, letters, and other second-hand studies were excluded.

After removing duplicates, the specific process is as follows: Two independent reviewers (YL and JT) checked the titles and abstracts to include and exclude irrelevant studies. The full texts are obtained and rechecked in more detail to finalize their inclusion. Any disagreement is resolved through discussion in which a third reviewer (JC) would participate. The final selection is checked and determined by a third reviewer (JC).

### Data extraction

The following items are extracted from each record: (1) publication details: title, first author, publishing year, unit, country, or region; (2) methodology details include: participants, disease types, diagnostic criteria, demographic characteristics (including age and gender), imaging modalities, data analysis strategies, interventions (including acupuncture and electroacupuncture). (3) Outcomes: significantly altered cerebral regions (defined by MNI/Talairach coordinates, cluster size, and statistical threshold), clinical assessment outcomes, and correlations between imaging and clinical data.

In this meta-analysis, team members extracted the participants whose neuroimaging data were analyzed for activation (which increased after the treatments in patients with musculoskeletal pain; POST > PRE), and deactivation (which decreased after the treatments in patients with musculoskeletal pain; PRE > POST) coordinates.

### Quality assessment

So far, there has been no standard checklist for quality assessment of individual functional neuroimaging studies. A checklist (Box 1) published in a previous meta-analysis was adopted (Li et al., 2019; Gong et al., 2020).

Box 1 **Quality assessment of individual studies.**

**Category 1: sample characteristics (10)**

1. Patients are evaluated with specific standardized diagnostic criteria (1).
2. Important demographic data (age and gender) are reported with mean (or median) and SDs (or range) (2).
3. Healthy control subjects are evaluated to exclude psychiatric and medical illnesses and demographic data are reported (1).
4. Important clinical variables (e.g., medication status, illness duration and severity) are reported with mean (or median) and SDs (or range) (4).
5. Sample size per group >10 (2).

**Category 2: methodology and reporting (10)**

6. Whole brain analysis is automated with no a priori regional selection (3).
7. Magnet strength at least 1.5T (1).
8. At least 5 min of resting state acquisition (1).
9. Whole brain coverage of resting scans (1).
10. The acquisition and preprocessing techniques are clearly described so that they can be reproduced (1).
11. Coordinates reported in a standard space (1).
12. Significant results are reported after correction for multiple testing using a standard statistical procedure (AlphaSim, FDR (False Discovery Rate), FWE (Family Wise Error) or permutation- based methods) (1).
13. Conclusions are consistent with the results obtained and the limitations are discussed (1).

### Activation likelihood estimation analysis

The researchers employed Ginger ALE version 3.0.2 (brainmap.org/ale) to conduct a neural coordinate-based activation likelihood estimation (ALE) meta-analysis on the neuroimaging data. Using Montreal Neuroimaging Institute (MNI) coordinates or converting them into an MNI-based coordinate system ensured the consistency of the coordinates. We used a cluster-level inference threshold correction algorithm for the ALE calculation, with *p* < 0.05 as the cluster-forming threshold and *p* < 0.05 for cluster-level inference. The number of permutations was 5000 for all calculations of simple ALE maps. We did not perform subgroup analyses because of the small number of included studies, foci, and patients.

## Results

### Search results

**Figure 1:**
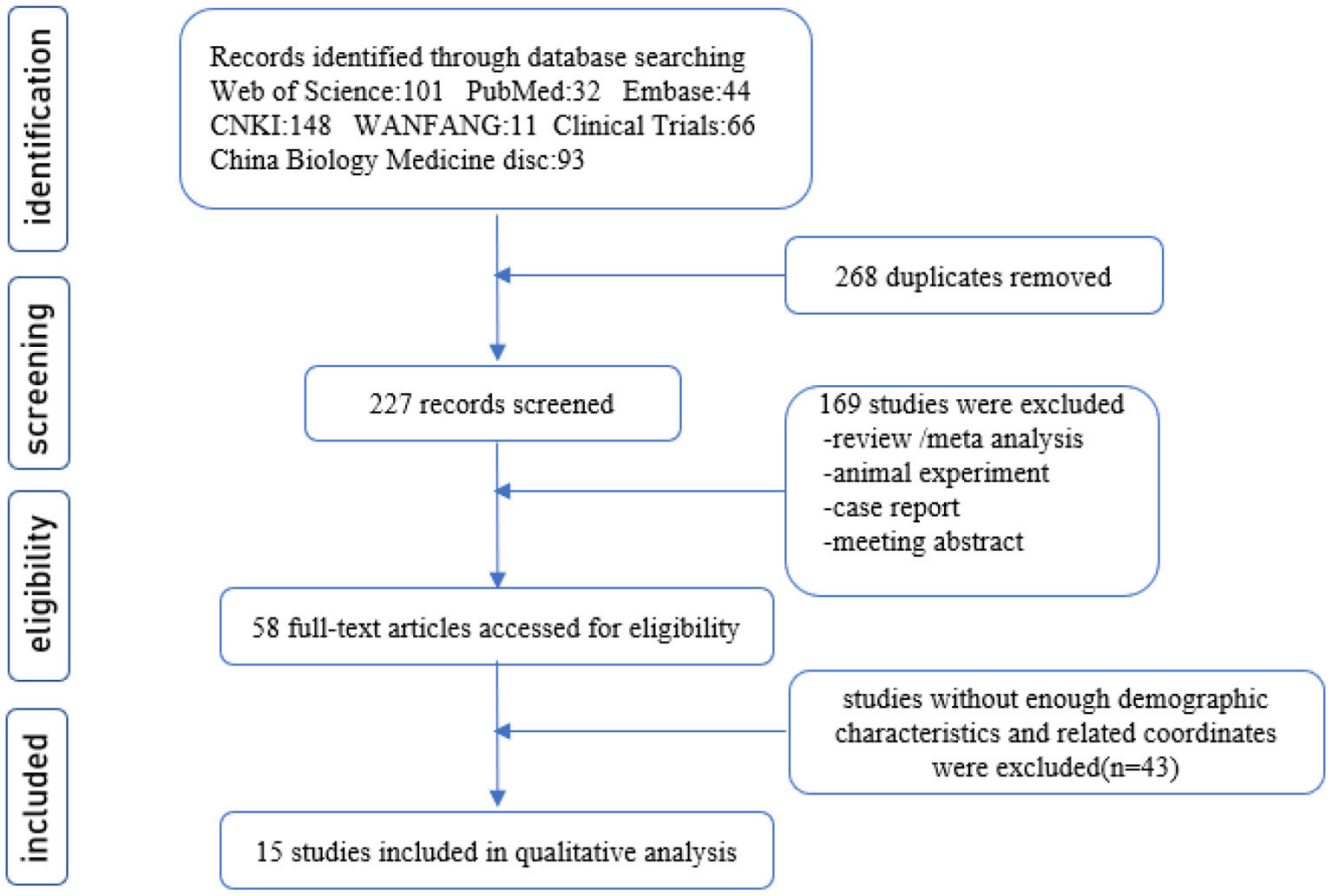
Flowchart shows the study selection process.

The flow diagram of the process depicting the literature search and study selection is shown in Figure 1. Among the 495 articles found in the literature search, 437 were excluded after reviewing the abstract, and another 43 were rejected after reviewing their full text.

Our research identified a total of 15 articles that assessed the effect of acupuncture for musculoskeletal pain on brain activity. All of these studies used functional magnetic resonance imaging (fMRI) scans. The studies reporting ALFF and ReHo as a measure of fMRI with the voxel-wise method of extracting image data were analyzed using ALE. All of them used FWE for multiple comparisons corrections (Table 2).

**Table 2:** Overview of the 15 studies included in the meta-analyses.

**Number**	**Study**	**Sample size (n)**	**Gender (M/F)**	**Age (years ±SD)**	**Reference space**	**Foci (n)**	**Threshold**
1	Guo et al. (2015)	MP30	14/16	NA	Talairach	10	*p* < 0.05 cor
2	Chen et al. (2014a)	MP15	7/8	NA	MNI	10	*p* < 0.05 cor
3	Zou et al. (2019)	MP32	15/17	46.371 ± 10.025	MNI	7	*p* < 0.05 cor
		HC25	12/13	40.014 ± 9.765			
4	Qu et al. (2021)	MP80	28/52	52.35 ± 4.62	MNI	5	*p* < 0.05 cor
		HC80	30/50	53.01 ± 4.58			
5	Liu et al. (2013)	MP15	9/6	25.7 ± 2.3	MNI	7	*p* < 0.05 cor
		HC15	9/6	25.7 ± 2.3			
6	Hou et al. (2014)	MP49	19/30	24.73 ± 1.46	MNI	10	*p* < 0.05 cor
		HC19	8/11	25.58 ± 3.32			
7	Chen et al. (2014b)	MP30	17/13	58 ± 8	MNI	4	*p* < 0.05 cor
8	Shi et al. (2015)	MP28	17/11	NA	MNI	69	*p* < 0.05 cor
		HC28	17/11	NA			
9	Zhang et al. (2018)	MP20	10/10	53.33 ± 5.26	MNI	10	*p* < 0.05 cor
10	Jian et al. (2018)	MP46	27/19	61.3 ± 6.9	MNI	7	*p* < 0.05 cor
11	Gollub et al. (2018)	MP43	17/26	57 ± 7	MNI	8	*p* < 0.05 cor
12	Xiang et al. (2019)	MP12	7/5	44.42 ± 6.99	MNI	5	*p* < 0.05 cor
13	Chen et al. (2015)	MP30	17/13	58 ± 8	MNI	22	*p* < 0.05 cor
14	Tu et al. (2019)	MP80	35/45	39.5 ± 13.0	MNI	4	*p* < 0.05 cor
		HC74	39/35	36.9 ± 8.2			
15	Napadow et al. (2012)	MP17	0/17	29.8 ± 4.0	MNI	5	*p* < 0.05 cor

*MP, musculoskeletal pain; HC, healthy control; NA, not available; M, male; F, female*.

### ALE results

**Figure 2:**
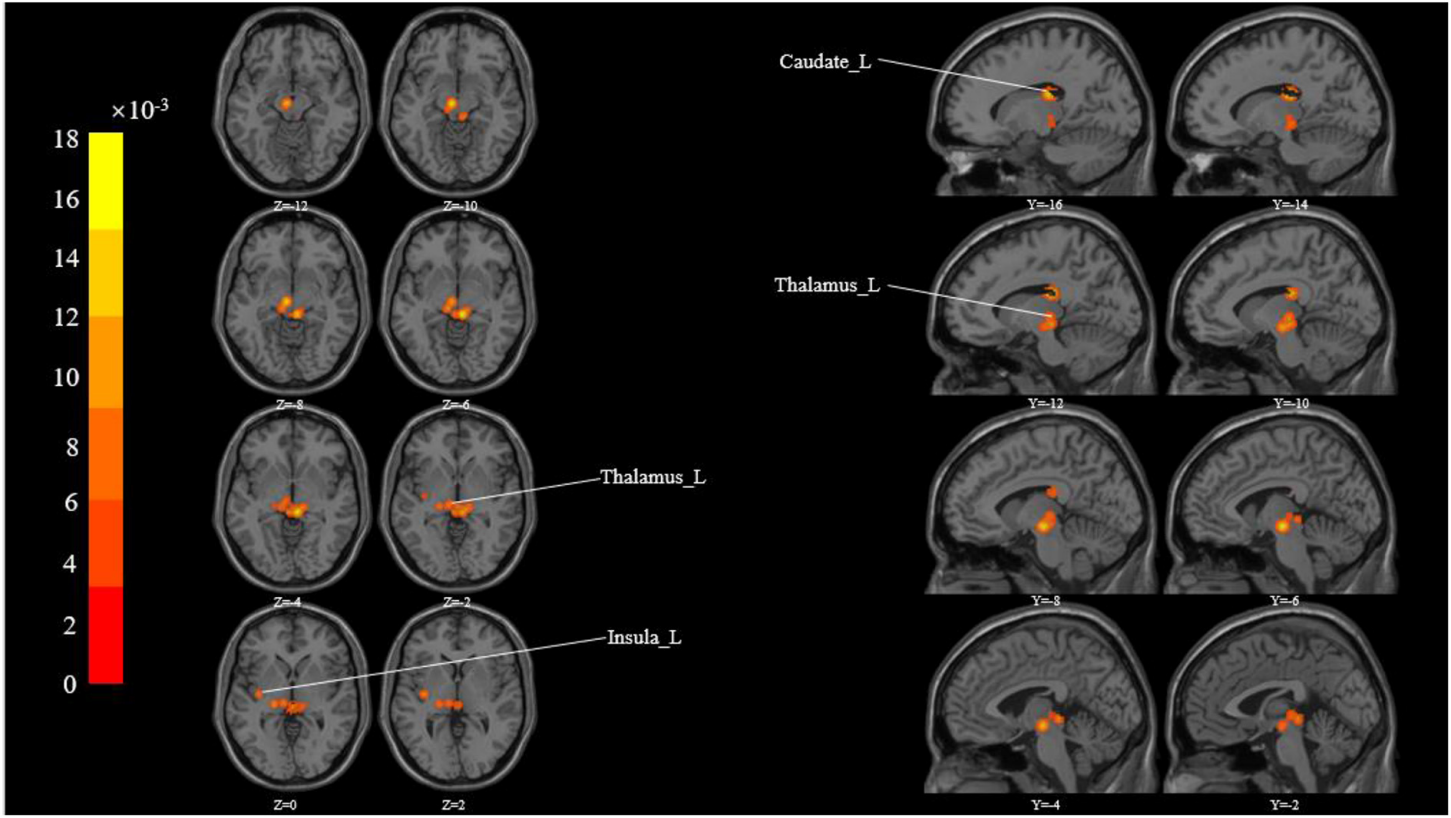
All activation likelihood estimate results for studies measuring. Cluster-level Inference P-FWE < 0.05, Permutations = 5000 Cluster-Forming P- Uncorrected < 0.05.

These studies yielded 183 foci of activation from 15 experiments. The ALE meta-analysis showed activated clusters in multiple cortical and sub-cortical brain structures in response to acupuncture across studies, including the thalamus, insula, caudate, claustrum, and lentiform nucleus (Table 3 and Figure 2).

**Table 3:** All clusters from the ALE analysis.

**Cluster #**	**x**	**y**	**z**	**ALE**	**P**	**Z**	**Label (nearest gray matter within 5 mm)**
1	6	−30	−6	0.018043537	6.81E-06	4.3498716	Right Cerebrum. Sub-lobar. Thalamus. Gray Matter.^*^.
1	−38	−12	16	0.01651409	2.30E-05	4.074941	Left Cerebrum. Sub-lobar. Insula. Gray Matter. Brodmann area 13
1	−6	−16	−10	0.016219338	2.91E-05	4.0203114	Left Brainstem. Midbrain.^*^. Gray Matter. Substania Nigra
1	−16	−22	22	0.016133353	3.08E-05	4.006909	Left Cerebrum. Sub-lobar. Caudate. Gray Matter. Caudate Tail
1	−10	−24	22	0.014161315	1.22E-04	3.6681178	Left Cerebrum. Sub-lobar. Thalamus. Gray Matter.^*^.
1	−32	0	24	0.01126301	6.64E-04	3.209974	Left Cerebrum. Sub-lobar. Insula. Gray Matter. Brodmann area 13
1	−32	−10	20	0.01083934	8.38E-04	3.142502	Left Cerebrum. Sub-lobar. Insula. Gray Matter. Brodmann area 13
1	−40	0	20	0.010651297	9.42E-04	3.1080759	Left Cerebrum. Sub-lobar. Insula. Gray Matter. Brodmann area 13
1	−12	−24	−6	0.010418649	0.001067337	3.0708263	Left Brainstem. Midbrain.^*^. Gray Matter. Substania Nigra
1	−22	−10	14	0.010235265	0.001180531	3.0406013	Left Cerebrum. Sub-lobar. Thalamus. Gray Matter. Ventral Lateral Nucleus
1	−2	−30	−4	0.010192102	0.001216393	3.0315785	Left Cerebrum. Sub-lobar. Thalamus. Gray Matter. Pulvinar
1	−10	−22	0	0.010163731	0.001239598	3.0258691	Left Cerebrum. Sub-lobar. Thalamus. Gray Matter. Mammillary Body
1	−20	−22	0	0.009802071	0.001657662	2.9368799	Left Cerebrum. Sub-lobar. Thalamus. Gray Matter. Ventral Posterior Lateral Nucleus
1	−36	−12	2	0.009715884	0.001762938	2.9177318	Left Cerebrum. Sub-lobar. Claustrum. Gray Matter.^*^.
1	−22	−18	10	0.009700557	0.001803456	2.9106383	Left Cerebrum. Sub-lobar. Thalamus. Gray Matter.^*^.

* *Clusters outside the brain atlas*.

## Discussion

This study aims to use voxel-based meta-analysis to identify brain regions commonly activated during acupuncture for musculoskeletal pain in various experimental paradigms. It is interesting to note that the ALE analysis method used in this study could help us to achieve reliable and strong results instead of a gamut of less reproducible findings from the individual studies. The ALE meta-analysis revealed activated clusters in multiple cortical and sub-cortical brain structures in response to acupuncture across studies, including Cerebrum (bilaterally), Left Brainstem (Sub-lobar and Midbrain), and Gyrus (Thalamus, Insula, Caudate, Claustrum, Lentiform Nucleus).

The ALE results suggest a core modulation brain region cluster for musculoskeletal pain treatment by acupuncture, which not only clarifies the pain perception of acupuncture intervention as well as the up and down stable pathways of pain modulation (cerebellum and brainstem) (Moulton et al., 2010; Ruscheweyh et al., 2014; Napadow et al., 2019; Mercer Lindsay et al., 2021) but more importantly, the subcortical pain matrix brain region was found to be involved in the central modulation of acupuncture for musculoskeletal pain, which is an important reference value for others to conduct the research on the central modulation of acupuncture for visceral pain.

Neuroimaging has identified a set of brain regions that respond to noxious stimuli by observing the brain's perception of injurious stimuli and pain modulation. And these regions are often referred to as the “pain matrix,” which includes the thalamus, anterior cingulate cortex, posterior cingulate cortex, insula, amygdala, primary and secondary somatosensory cortex (S1 and S2), and periaqueductal gray matter (Melzack, 1999; Apkarian et al., 2005; May, 2007). Among them, the thalamus and insula play an extremely important role in pain perception and pain processing. The activation of the thalamus is mainly connected with the first-order processing of sensory information. It receives signals from its periphery and sends them to the hypothalamus, insula, motor, and somatosensory cortex (Al-Chaer et al., 1996; Aziz et al., 2000; Olesen et al., 2016; Lee et al., 2019). It was found that the post-effects of acupuncture can cause changes in functional connectivity between important brain regions in the pain matrix, exerting the analgesic effects of acupuncture by decreasing the thalamus-anterior cingulate pain upload pathway and strengthening the ventral medial prefrontal-anterior cingulate descending inhibitory pathway (Roy et al., 2012). There is significant evidence that the insula plays a critical role in pain processing, which could cause pain perception after acupuncture intervention and integrate sensory information from visceral and motor activity with limbic system input (Moisset et al., 2010; Olesen et al., 2016; Lee et al., 2019). The activation of the insula indicates that in the acupuncture state, the brain accelerates the processing of pain information, carries out sensory integration more efficiently, provides timely feedback to the various stress systems of the organism, and speeds up the processing of pain stimulation so as to better relieve pain (Zhang et al., 2014). The ALE results also found a significant increase in activity in regions including the caudate, claustrum, and lentiform nucleus on fMRI scans following acupuncture. These regions are the main components of the basal ganglia (Graybiel, 2005; Kreitzer and Malenka, 2008; Yelnik, 2008). The function of the basal ganglia is to control autonomous movement and participate in advanced cognitive functions such as memory, emotion, and reward learning (Herrero et al., 2002; Nagy et al., 2006; Draganski et al., 2008). Previous studies have shown that the basal ganglia may be involved in most aspects of pain processing, including the cognitive dimension of pain and pain modulation (Chudler and Dong, 1995). Also, adequate modulation of the basal ganglia subregions may be related to autonomic dyskinesia caused by musculoskeletal pain (Borsook et al., 2010b). The combination of the above findings provides some support for the evidence of acupuncture's effectiveness in treating musculoskeletal pain diseases.

In general, up till now, a large number of neuroimaging studies have shown that patients with musculoskeletal pain exhibit structural and functional changes in brain regions. The majority of those regions are associated with multiple aspects of pain processing. Specifically, complex neuronal network interactions in the organism are required to form pain perception (Cai et al., 2018). When pain strikes, the brain temporarily and dynamically integrates multiple brain regions to process pain information. The “pain matrix” summarizes these brain regions involved in the process of pain. The brainstem, prefrontal, thalamus, insula, cingulate gyrus, subcortical areas, and somatosensory cortex are all part of this matrix, which is responsible for sensory, emotional, and cognitive functions (Apkarian et al., 2005). In this study, activated signals in some brain regions, such as the caudate, claustrum, and lentiform nucleus, were different from the healthy controls. Previous studies also present the potential for alterations in these brain regions. The cerebral cortex's function as an essential component of the pain modulation system has received widespread attention (Ong et al., 2019). We think that the thalamus, insula, caudate, claustrum, and lentiform nucleus are crucial to the acupuncture mechanism previous studies have found that the effect of acupuncture is to elevate mechanical pain thresholds, change signaling levels in several important pain pathway areas, and have positive impacts on a variety of pain syndromes and states (Baeumler et al., 2014). To truly obtain sufficient evidence of acupuncture's effectiveness in treating musculoskeletal pain diseases, a greater homogeneity of the different study populations, experimental paradigms, and data analysis pipelines must be sought. The complexity of musculoskeletal pain diseases was a prevalent concern in the process of including the literature, and we repeated the ALE analysis after excluding studies with sample sizes <10 to remove the bias introduced by small-study effects. It was also specified that the included studies had to be fMRI whole-brain analyses. Although the paradigms in our study were not identical, the approach to functional neuroimaging techniques was similar enough across studies to warrant comparison. In this study, ALE analysis was used to determine the probability of brain regions being activated or deactivated by integrating the study coordinates of all screened studies. The results provided feedback information for the mechanisms of brain function with acupuncture for musculoskeletal pain. Furthermore, evaluating the study results may indicate the considerable modulatory effects of acupuncture for musculoskeletal pain, which is consistent with the “pain matrix” theory. These findings provide various new insights into the processes of acupuncture for musculoskeletal pain and a possible explanation for the therapy's clinical efficacy.

### Limitations

Subjects included in the analysis came from heterogeneous musculoskeletal pain disorders. Consequently, our findings merely give a glimpse into the mechanism of acupuncture's effect on musculoskeletal pain.

## Conclusion

The ALE meta-analysis revealed activated clusters in multiple cortical and sub-cortical brain structures, especially basal ganglia, in response to acupuncture across studies.

## Data availability statement

The original contributions presented in the study are included in the article/supplementary material, further inquiries can be directed to the corresponding author/s.

## Author contributions

GH drafted the manuscript and designed the study under the guidance of LL and FZ. GH and ZT performed the study extraction and meta-analysis. JC, SW, AL, and NL helped in literature search and data analyses. YL and JT offered good suggestions. LL and FZ revised the manuscript. All authors contributed toward revising the manuscript and gave the final approval of the version to be published.

## Funding

This study was supported by the National Key R&D Program of China (No. 2018YFC1704606) and the Key R&D Program of Sichuan Provincial Department of Science and Technology (No. 2022YFS0401).

## Conflict of interest

The authors declare that the research was conducted in the absence of any commercial or financial relationships that could be construed as a potential conflict of interest.

## Publisher's note

All claims expressed in this article are solely those of the authors and do not necessarily represent those of their affiliated organizations, or those of the publisher, the editors and the reviewers. Any product that may be evaluated in this article, or claim that may be made by its manufacturer, is not guaranteed or endorsed by the publisher.

## References

[B1] Abdulmonem A. Hanan A. Elaf A. Haneen T. Jenan A. (2014). The prevalence of musculoskeletal pain & its associated factors among female Saudi school teachers. Pak. J. Med. Sci. 30, 1191–1196. 10.12669/pjms.306.5778 25674106 PMC4320698

[B2] Al-Chaer E. D. Lawand N. B. Westlund K. N. Willis W. D. (1996). Visceral nociceptive input into the ventral posterolateral nucleus of the thalamus: a new function for the dorsal column pathway. J. Neurophysiol. 76, 2661–2674. 10.1152/jn.1996.76.4.2661 8899636

[B3] Apkarian A. V. Bushnell M. C. Treede R. D. Zubieta J. K. (2005). Human brain mechanisms of pain perception and regulation in health and disease. Eur. J. Pain 9, 463–484. 10.1016/j.ejpain.2004.11.001 15979027

[B4] Aziz Q. Schnitzler A. Enck P. (2000). Functional neuroimaging of visceral sensation. J. Clin. Neurophysiol. 17, 604–612. 10.1097/00004691-200011000-00006 11151978

[B5] Baeumler P. I. Fleckenstein J. Takayama S. Simang M. Seki T. Irnich D. (2014). Effects of acupuncture on sensory perception: a systematic review and meta-analysis. PLoS ONE 9:e113731. 10.1371/journal.pone.0113731 25502787 PMC4264748

[B6] Borsook D. Sava S. Becerra L. (2010a). The pain imaging revolution: advancing pain into the 21st century. Neuroscientist 16, 171–185. 10.1177/1073858409349902 20400714 PMC3370428

[B7] Borsook D. Upadhyay J. Chudler E. H. Becerra L. (2010b). A key role of the basal ganglia in pain and analgesia–insights gained through human functional imaging. Mol. Pain 6, 27. 10.1186/1744-8069-6-27 20465845 PMC2883978

[B8] Cai R. L. Shen G. M. Wang H. Guan Y. Y. (2018). Brain functional connectivity network studies of acupuncture: a systematic review on resting-state fMRI. J. Integr. Med. 16, 26–33. 10.1016/j.joim.2017.12.002 29397089

[B9] Chae Y. Chang D. S. Lee S. H. Jung W. M. Lee I. S. Jackson S. . (2013). Inserting needles into the body: a meta-analysis of brain activity associated with acupuncture needle stimulation. J. Pain 14, 215–222. 10.1016/j.jpain.2012.11.011 23395475

[B10] Chen J. Liu G. Chen J. Liu X. Liu X. Ling Y. (2014a). Resting state fMRI study of different acupuncture analgesia therapies. Chin. J. Gerontol. 33, 2977–2979. 10.1155/2021/6616060 33859708 PMC8009717

[B11] Chen X. Spaeth R. B. Freeman S. G. Scarborough D. M. Hashmi J. A. Wey H. Y. . (2015). The modulation effect of longitudinal acupuncture on resting state functional connectivity in knee osteoarthritis patients. Mol Pain 11, 67. 10.1186/s12990-015-0071-9 26511911 PMC4625557

[B12] Chen X. Spaeth R. B. Retzepi K. Ott D. Kong J. (2014b). Acupuncture modulates cortical thickness and functional connectivity in knee osteoarthritis patients. Sci. Rep. 4, 6482. 10.1038/srep06482 25258037 PMC4175730

[B13] Chudler E. H. Dong W. K. (1995). The role of the basal ganglia in nociception and pain. Pain 60, 3–38. 10.1016/0304-3959(94)00172-B 7715939

[B14] Draganski B. Kherif F. Klöppel S. Cook P. A. Alexander D. C. Parker G. J. . (2008). Evidence for segregated and integrative connectivity patterns in the human Basal Ganglia. J. Neurosci. 28, 7143–7152. 10.1523/JNEUROSCI.1486-08.2008 18614684 PMC6670486

[B15] Farmer M. A. Baliki M. N. Apkarian A. V. (2012). A dynamic network perspective of chronic pain. Neurosci. Lett. 520, 197–203. 10.1016/j.neulet.2012.05.001 22579823 PMC3377811

[B16] Garcia-Larrea L. Peyron R. (2013). Pain matrices and neuropathic pain matrices: a review. Pain 154(Suppl 1), S29–s43. 10.1016/j.pain.2013.09.001 24021862

[B17] Gollub R. L. Kirsch I. Maleki N. Wasan A. D. Edwards R. R. Tu Y. . (2018). A functional neuroimaging study of expectancy effects on pain response in patients with knee osteoarthritis. J. Pain. 19, 515–527. 10.1016/j.jpain.2017.12.260 29325883 PMC5927817

[B18] Gong J. Wang J. Luo X. Chen G. Huang H. Huang R. . (2020). Abnormalities of intrinsic regional brain activity in first-episode and chronic schizophrenia: a meta-analysis of resting-state functional MRI. J. Psychiatry Neurosci. 45, 55–68. 10.1503/jpn.180245 31580042 PMC6919918

[B19] Graybiel A. M. (2005). The basal ganglia: learning new tricks and loving it. Curr. Opin. Neurobiol. 15, 638–644. 10.1016/j.conb.2005.10.006 16271465

[B20] Guo T. Xiao S. Chu W. Li M. (2015). Changes of different analgesic acupuncture treatments for regional homogeneity of brain activity in resting state by functional magnetic resonance imaging and the similarities and differences between their mechanisms. J. Clin. Acupunct. Moxibust. 31, 25–27.

[B21] Henry D. E. Chiodo A. E. Yang W. (2011). Central nervous system reorganization in a variety of chronic pain states: a review. PMR 3, 1116–1125. 10.1016/j.pmrj.2011.05.018 22192321

[B22] Herrero M. T. Barcia C. Navarro J. M. (2002). Functional anatomy of thalamus and basal ganglia. Childs. Nerv. Syst. 18, 386–404. 10.1007/s00381-002-0604-1 12192499

[B23] Hou X. Chen W. Chen J. Zhang D. Liu X. Liu B. (2014). The study of regional homogeneity of DMN in patients with CSNP after acupuncture in group acupoint. Chin. J. Magn. Resonan. Imag. 5, 436–440.

[B24] Jian K. Zengjian W. Jaclyn L. Domenic M. Robert E. Irving K. . (2018). Enhancing treatment of osteoarthritis knee pain by boosting expectancy: A functional neuroimaging study. NeuroImage Clin. 18, 325–344. 10.1016/j.nicl.2018.01.021 29868449 PMC5984593

[B25] Kreitzer A. C. Malenka R. C. (2008). Striatal plasticity and basal ganglia circuit function. Neuron 60, 543–554. 10.1016/j.neuron.2008.11.005 19038213 PMC2724179

[B26] Lawrence R. C. Helmick C. G. Arnett F. C. Deyo R. A. Felson D. T. Giannini E. H. . (1998). Estimates of the prevalence of arthritis and selected musculoskeletal disorders in the United States. Arthritis Rheum 41, 778–799. 10.1002/1529-0131(199805)41:5<778::AID-ART4>3.0.CO;2-V 9588729

[B27] Lee I. S. Cheon S. Park J. Y. (2019). Central and peripheral mechanism of acupuncture analgesia on visceral pain: a systematic review. Evid. Based Complement. Alternat. Med. 2019:1304152. 10.1155/2019/1304152 31186654 PMC6521529

[B28] Lenoir D. De Pauw R. Van Oosterwijck S. Cagnie B. Meeus M. (2020). Acupuncture versus sham acupuncture: a meta-analysis on evidence for longer-term effects of acupuncture in musculoskeletal disorders. Clin. J. Pain 36, 533–549. 10.1097/AJP.0000000000000812 32028381

[B29] Li S. Hu N. Zhang W. Tao B. Dai J. Gong Y. . (2019). Dysconnectivity of multiple brain networks in schizophrenia: a meta-analysis of resting-state functional connectivity. Front. Psychiatry 10, 482. 10.3389/fpsyt.2019.00482 31354545 PMC6639431

[B30] Liu Z. Wu W. Zhang S. Guo S. Yang J. (2013). Pain matrix response to acupuncture stimuli in individuals with acute low back pain: an fmri study. Chin. J. Pain Med. 19, 201–205.

[B31] May A. (2007). Neuroimaging: visualising the brain in pain. Neurol Sci 28(Suppl 2), S101–107. 10.1007/s10072-007-0760-x 17508154

[B32] Melhorn J. M. (2014). Epidemiology of musculoskeletal disorders and workplace factors, in: Handbook of Musculoskeletal Pain and Disability Disorders in the Workplace, eds Gatchel R. J. Schultz I. Z. (New York, NY: Springer New York), 175–204. 10.1007/978-1-4939-0612-3_10

[B33] Melzack R. (1999). From the gate to the neuromatrix. Pain Suppl. 6, S121–s126. 10.1016/S0304-3959(99)00145-1 10491980

[B34] Mercer Lindsay N. Chen C. Gilam G. Mackey S. Scherrer G. (2021). Brain circuits for pain and its treatment. Sci. Transl. Med. 13, eabj7360. 10.1126/scitranslmed.abj7360 34757810 PMC8675872

[B35] Mitsi V. Zachariou V. (2016). Modulation of pain, nociception, and analgesia by the brain reward center. Neuroscience 338, 81–92. 10.1016/j.neuroscience.2016.05.017 27189881 PMC5083150

[B36] Moisset X. Bouhassira D. Denis D. Dominique G. Benoit C. Sabat,é J. M. (2010). Anatomical connections between brain areas activated during rectal distension in healthy volunteers: a visceral pain network. Eur. J. Pain 14, 142–148. 10.1016/j.ejpain.2009.04.011 19473859

[B37] Moulton E. A. Schmahmann J. D. Becerra L. Borsook D. (2010). The cerebellum and pain: passive integrator or active participator? Brain Res. Rev. 65, 14–27. 10.1016/j.brainresrev.2010.05.005 20553761 PMC2943015

[B38] Mu J. Furlan A. D. Lam W. Y. Hsu M. Y. Ning Z. Lao L. (2020). Acupuncture for chronic nonspecific low back pain. Cochrane Database Syst. Rev. 12, Cd013814. 10.1002/14651858.CD013814 33306198 PMC8095030

[B39] Nagy A. Eördegh G. Paróczy Z. Márkus Z. Benedek G. (2006). Multisensory integration in the basal ganglia. Eur. J. Neurosci. 24, 917–924. 10.1111/j.1460-9568.2006.04942.x 16930419

[B40] Napadow V. Kim J. Clauw D. J. Harris R.E. (2012). Decreased intrinsic brain connectivity is associated with reduced clinical pain in fibromyalgia. Arthritis Rheum. 64, 2398–2403. 10.1002/art.34412 22294427 PMC3349799

[B41] Napadow V. Sclocco R. Henderson L. A. (2019). Brainstem neuroimaging of nociception and pain circuitries. Pain Rep 4, e745. 10.1097/PR9.0000000000000745 31579846 PMC6727990

[B42] Olesen A. E. Farmer A. D. Olesen S. S. Aziz Q. Drewes A. M. (2016). Management of chronic visceral pain. Pain Manag. 6, 469–486. 10.2217/pmt-2015-0011 27256577

[B43] Ong W. Y. Stohler C. S. Herr D. R. (2019). Role of the Prefrontal Cortex in Pain Processing. Mol. Neurobiol. 56, 1137–1166. 10.1007/s12035-018-1130-9 29876878 PMC6400876

[B44] Qaseem A. Wilt T. J. McLean R. M. Forciea M. A. Denberg T. D. Barry M. J. . (2017). noninvasive treatments for acute, subacute, and chronic low back pain: a clinical practice guideline from the American College of Physicians. Ann. Intern. Med. 166, 514–530. 10.7326/M16-2367 28192789

[B45] Qu B. Wang H. Zhao C. Shi G. (2021). Clinical efficacy evaluation and central mechanism study of acupuncture in treating chronic knee osteoarthritis. J. Xinjiang Med. Univ. 44, 600–604.

[B46] Roy M. Shohamy D. Wager T. D. (2012). Ventromedial prefrontal-subcortical systems and the generation of affective meaning. Trends Cogn. Sci. 16, 147–156. 10.1016/j.tics.2012.01.005 22310704 PMC3318966

[B47] Ruscheweyh R. Kühnel M. Filippopulos F. Blum B. Eggert T. Straube A. (2014). Altered experimental pain perception after cerebellar infarction. Pain 155, 1303–1312. 10.1016/j.pain.2014.04.006 24721690

[B48] Shi Y. Liu Z. Zhang S. Li Q. Guo S. Yang J. . (2015). Brain network response to acupuncture stimuli in experimental acute low back pain: An fMRI Study. Evid. Based Complement Alternat. Med. 2015, 210120. 10.1155/2015/210120 26161117 PMC4487721

[B49] Skootsky S. A. Jaeger B. Oye R. K. (1989). Prevalence of myofascial pain in general internal medicine practice. West. J. Med. 151, 157–160. 2788962 PMC1026905

[B50] Tu Y. Jung M. Gollub R. L. Napadow V. Gerber J. Ortiz A. . (2019). Abnormal medial prefrontal cortex functional connectivity and its association with clinical symptoms in chronic low back pain. Pain 160, 1308–1318. 10.1097/j.pain.0000000000001507 31107712 PMC6530583

[B51] Ussai S. Miceli L. Pisa F. E. Bednarova R. Giordano A. Della Rocca G. . (2015). Impact of potential inappropriate NSAIDs use in chronic pain. Drug Des. Devel. Ther. 9, 2073–2077. 10.2147/DDDT.S80686 25926717 PMC4403601

[B52] Villarreal Santiago M. Tumilty S. Macznik A. Mani R. (2016). Does acupuncture alter pain-related functional connectivity of the central nervous system? A systematic review. J. Acupunct. Meridian. Stud. 9, 167–177. 10.1016/j.jams.2015.11.038 27555221

[B53] Wolfe F. Ross K. Anderson J. Russell I. J. Hebert L. (1995). The prevalence and characteristics of fibromyalgia in the general population. Arthritis Rheum. 38, 19–28. 10.1002/art.1780380104 7818567

[B54] Xiang A. Yu Y. Jia X. Ma H. Liu H. Zhang Y. . (2019). The low-frequency BOLD signal oscillation response in the insular associated to immediate analgesia of ankle acupuncture in patients with chronic low back pain. J. Pain Res. 12, 841–850. 10.2147/JPR.S189390 30881095 PMC6400126

[B55] Yelnik J. (2008). Modeling the organization of the basal ganglia. Rev. Neurol. (Paris) 164, 969–976. 10.1016/j.neurol.2008.04.019 18808769

[B56] Yuan Q. L. Wang P. Liu L. Sun F. Cai Y. S. Wu W. T. . (2016). Acupuncture for musculoskeletal pain: A meta-analysis and meta-regression of sham-controlled randomized clinical trials. Sci. Rep. 6, 30675. 10.1038/srep30675 27471137 PMC4965798

[B57] Zhang S. Wang X. Yan C. Q. Hu S. Q. Huo J. W. Wang Z. Y. . (2018). Different mechanisms of contralateral- or ipsilateral-acupuncture to modulate the brain activity in patients with unilateral chronic shoulder pain: a pilot fMRI study. J. Pain Res. 11, 505–514. 10.2147/JPR.S152550 29563830 PMC5846304

[B58] Zhang S. S. Wu W. Liu Z. P. Huang G. Z. Guo S. G. Yang J. M. (2014). Altered regional homogeneity in experimentally induced low back pain: a resting-state fMRI study. J. Neuroeng. Rehabil. 11, 115. 10.1186/1743-0003-11-115 25080831 PMC4237877

[B59] Zhang Y. Wang C. (2020). Acupuncture and Chronic Musculoskeletal Pain. Curr. Rheumatol. Rep. 22, 80. 10.1007/s11926-020-00954-z 32978666 PMC8719359

[B60] Zou Y. Tang W. Wang S. Huang J. Li J. (2019). Objective evaluation on brain network imaging of treating same disease with different methods effect of acupuncture for chronic low back pain (cLBP). Fudan Univ. J. Med. Sci. 46, 167–173. 10.3969/j.issn.1672-8467.2019.02.004

