# Supplementary material for: Coordinate-based (ALE) meta-analysis of acupuncture for musculoskeletal pain
Source: Front Neurosci. 2022 Jul 22;16:906875. doi: 10.3389/fnins.2022.906875 (PMC9354890; doi:10.3389/fnins.2022.906875)
Supplement: Supplementary file 1 [file Table_1.pdf]

PRISMA-A checklist

Subjects	PRISMA for Acupuncture
Title	
**Title**	1* Identify the report as a systematic review, meta-analysis, or both; if applicable, state the specific type of acupuncture treatment, such as manual acupuncture or electroacupuncture.<br>Coordinate-Based (ALE) Meta-Analysis of Acupuncture for Musculoskeletal Pain
Abstract	
**Structured summary**	2† Provide a structured summary including, as applicable: background; objectives; data sources; study eligibility criteria, participants, and interventions; study appraisal and synthesis methods; results limitations; conclusions and implications of key findings; systematic review registration number.<br>**Background:** Neuroimaging studies have been widely used to investigate brain regions' alterations in musculoskeletal pain patients. However, inconsistent results have hindered our understanding of the central modulatory effects of acupuncture for musculoskeletal pain. The main objective of our investigation has been to obtain comprehensive evidence of acupuncture for musculoskeletal pain diseases.<br>**Methods:** The PubMed, Web of Science, Google Scholar, Embase, China National Knowledge Infrastructure (CNKI), VIP Database, China Biology Medicine disc Database, Clinical Trial Registration Platform, and Wanfang Database were searched for neuroimaging studies on musculoskeletal pain diseases published from inception up to November 2021. The present meta-analysis was registered in PROSPERO (no. CRD42021227850).<br>**Results:** A total of 15 neuroimaging studies with 183 foci of activation were included in this study. The ALE meta-analysis revealed activated clusters in multiple cortical and sub-cortical brain structures in response to acupuncture across studies, including the thalamus, insula, caudate, claustrum, and lentiform nucleus.<br>**Limitations:** Subjects included in the analysis came from heterogeneous musculoskeletal pain disorders. Consequently, our findings merely give a glimpse into the mechanism of acupuncture's effect on musculoskeletal pain.<br>**Conclusions:** The studies showed that acupuncture could modulate different brain regions, including the thalamus, insula, caudate, claustrum, and lentiform nucleus. The findings offer several insights into the potential mechanisms of acupuncture for musculoskeletal pain and provide a possible explanation for the observed clinical benefit of this therapy.

Introduction	
**Rationale**	3* Describe the rationale for what is already known about acupuncture for the target condition in the background; if applicable, state what is already known about the specific types of acupuncture to be studied, and describe whether there is any difference of the effects among different types of acupuncture.<br>As an efficacious treatment for musculoskeletal pain, acupuncture has a long history of relieving the symptoms of such diseases in the East (Lenoir et al., 2020). It has been recommended as a positive therapy in a clinical practice guideline from the American College of Physicians (Qaseem et al., 2017). Acupuncture has been used to treat many types of musculoskeletal pain diseases (Mu et al., 2020; Zhang and Wang, 2020). In previous studies, acupuncture has been found to regulate abnormal neural activities of the "pain matrix," mainly the second and third-order matrices responsible for pain memory in musculoskeletal pain sufferers (Apkarian et al., 2005; Borsook et al., 2010a; Garcia-Larrea and Peyron, 2013)
Methods	
**Eligibility criteria**	6† Specify study characteristics (e.g., PICOS, length of follow-up) and report characteristics (e.g., years considered, language, publication status) used as criteria for eligibility, giving rationale.<br>6a‡ Describe the diagnostic criteria of the target condition in Western medicine.<br>The diagnosis of neck pain and low back pain were defined according to the criteria of the North American Spine Society.<br>The diagnosis of fibromyalgia was defined according to the criteria of the American Rheumatism Association<br>6b‡ Describe the types of acupuncture to be included, such as traditional acupuncture, electroacupuncture, or fire acupuncture.<br>We included both traditional acupuncture, in which the needles are inserted in classical meridian points, and contemporary acupuncture, in which the needles are inserted in non-meridian or trigger points, regardless of the source of stimulation (for example, scalp acupuncture, wrist-ankle needle or electrical stimulation, etc.)
**Information sources**	7* Describe all sources of information (e.g., databases with dates of coverage, contact with study authors to identify additional studies) in the search, and report the date of the last search. If applicable, report the databases or complementary search methods for acupuncture or traditional medicine.<br>*Studies were obtained from the following databases: The PubMed, Web of Science, Google Scholar, Embase, China National Knowledge Infrastructure (CNKI), VIP Database, China Biology Medicine disc Database, Clinical Trial Registration Platform, and Wanfang*

	Database were searched for neuroimaging studies on musculoskeletal pain diseases published from inception up to November 2021. The relevant references from the retrieved papers have been added to the database for this study.
**Search**	8[*] Present full electronic search strategy for at least one commonly used database (e.g. MEDLINE), including any limits used, such that it could be repeated. If applicable, include the full search strategy for at least a Western and a traditional medicine database for each systematic review where both were used.<br>PubMed searching strategy<br>#1 musculoskeletal pain (MeSH Terms);#2 musculoskeletal pain (All Fields);#3 musculoskeletal disease (All Fields);#4musculoskeletal disorders (All Fields);#5muscular diseases (All Fields);#6chronic musculoskeletal pain (All Fields);#7musculoskeletal Conditions (All Fields);#8muscle pain (All Fields);#9Myalgia (All Fields);#10myofascial Pain (All Fields);#11Fibromyalgia (MeSH Terms);#12neck pain (MeSH Terms);#13Osteoarthritis (MeSH Terms);#14Arthritis (MeSH Terms);#15Arthrosis (MeSH Terms);#16Arthralgia (MeSH Terms);#17Joint Diseases (MeSH Terms);#18Low Back Pain (MeSH Terms);#19Lumbago (MeSH Terms);#20Back Pain (MeSH Terms);#21Backache (MeSH Terms);#22Shoulder Pain (MeSH Terms);#23Cervicalgia (MeSH Terms);#24#1OR#2 OR #3 OR #4 OR #5 OR #6OR#7OR #8 OR #9 OR #10 OR #11 OR#12 OR#13OR #14 OR#15 OR #16 OR #17 OR #18 OR #19 OR #20 OR #21 OR #22 OR #23;#25 acupuncture (MeSH Terms);#26 acupuncture Therapy (MeSH Terms);#27 acupoint (MeSH Terms);#28 acupuncture Point (MeSH Terms);#29 electroacupuncture (MeSH Terms);#30 electro-acupuncture (MeSH Terms);#31#25OR#26 OR#27 OR#28 OR#29 OR#30;#32Functional magnetic resonance imaging (MeSH Terms);#33 Functional MRI (MeSH Terms);#34 fMRI (MeSH Terms);#35#32 OR#33 OR#34;#36 Final search terms: #24AND #32 AND #35
**Data items**	11[*] List and define all variables for which data were sought (e.g., PICOS, funding sources) and any assumptions and simplifications made; describe data items about details of acupuncture interventions and controls (**e.g.,** sham acupuncture) referring to TIDieR when applicable.<br>We recorded the following items: (1) publication details: title, first author, publishing year, unit, country, or region; (2) methodology details include: participants, disease types, diagnostic criteria, demographic characteristics (including age and gender), imaging modalities, data analysis strategies, interventions (including acupuncture and electroacupuncture). (3) Outcomes: significantly altered cerebral regions (defined by MNI/Talairach coordinates, cluster size, and statistical threshold), clinical assessment outcomes, and correlations between imaging and clinical data.

Results	
	18* For each study, present characteristics that were extracted (e.g., study size, PICOS, follow-up period) and provide the citations of the included studies. Summarize details of the acupuncture intervention for each study in a table referring to TIDieR.
**Study characteristics**	Interventions — ACUPUNCTURE<br>Number allocated to acupuncture: not reported<br>Style of acupuncture: Chinese<br>Points selection: Formula<br>Points stimulated: Names of points were not reported<br>Insertion depth: Not stated<br>Method of stimulation: Not reported<br><br>CONTROL GROUP A INTERVENTION:Sham acupuncture<br>Number allocated to control group : not reported<br>Points selection:Formula<br>Points stimulated: Names of points were not stated<br>Insertion depth: Not stated<br>Method of stimulation: None<br>Any co-interventions in all groups? No

Discussion	
**Limitations**	25[†] Discuss limitations at study and outcome level (e.g., risk of bias), and at review level (e.g., incomplete retrieval of identified research, reporting bias).<br>Subjects included in the analysis came from heterogeneous musculoskeletal pain disorders. Consequently, our findings merely give a glimpse into the mechanism of acupuncture's effect on musculoskeletal pain.
**Conclusions**	26[†] Provide a general interpretation of the results in the context of other evidence, and implications for future research.<br>The ALE meta-analysis revealed activated clusters in multiple cortical and sub-cortical brain structures, especially basal ganglia, in response to acupuncture across studies.
Funding	
**Funding**	27[†] Describe sources of funding for the systematic review and other support (e.g., supply of data); role of funders for the systematic review.<br>This study was supported by the National Key R&D Program of China (No. 2018YFC1704606) and the Key R&D Program of Sichuan Provincial Department of Science and Technology (No. 2022YFS0401).

Note: * modified original item [†] unmodified item from PRISMA [‡] new extended item

References:

Apkarian, A.V., Bushnell, M.C., Treede, R.D., and Zubieta, J.K. (2005). Human brain mechanisms of pain perception and regulation in health and disease. *Eur J Pain* 9(4)**,** 463-484. doi: 10.1016/j.ejpain.2004.11.001

Borsook, D., Sava, S., and Becerra, L. (2010a). The pain imaging revolution: advancing pain into the 21st century. *Neuroscientist* 16(2)**,** 171-185. doi: 10.1177/1073858409349902

Garcia-Larrea, L., and Peyron, R. (2013). Pain matrices and neuropathic pain matrices: a review. *Pain* 154 Suppl 1**,** S29-s43. doi: 10.1016/j.pain.2013.09.001

Lee, I.S., Cheon, S., and Park, J.Y. (2019). Central and Peripheral Mechanism of Acupuncture Analgesia on Visceral Pain: A Systematic Review. *Evid Based Complement Alternat Med* 2019**,** 1304152. doi: 10.1155/2019/1304152.

Mu, J., Furlan, A.D., Lam, W.Y., Hsu, M.Y., Ning, Z., and Lao, L. (2020). Acupuncture for chronic nonspecific low back pain. *Cochrane Database Syst Rev* 12(12)**,** Cd013814. doi: 10.1002/14651858.Cd013814

Qaseem, A., Wilt, T.J., McLean, R.M., Forciea, M.A., Denberg, T.D., Barry, M.J., et al. (2017). Noninvasive Treatments for Acute, Subacute, and Chronic Low Back Pain: A Clinical Practice Guideline From the American College of Physicians. *Ann Intern Med* 166(7)**,** 514-530. doi: 10.7326/m16-2367.
